# Level of development, foreign direct investment and domestic investment in food manufacturing

**DOI:** 10.12688/f1000research.28681.3

**Published:** 2022-07-01

**Authors:** Justice Gameli Djokoto

**Affiliations:** 1 Agribusiness Management Department, Central University, Ghana, Accra, Greater Accra Region, +233, Ghana

**Keywords:** Crowding-in, crowding-out, developing countries, developed countries, food manufacturing, foreign direct investment, domestic investment.

## Abstract

**Background:** Whilst the literature on the complementarity and substitutability of foreign direct investment (FDI) on domestic investment (DI) is not uncommon, the facet of food manufacturing is non-existent. This paper fills this void by investigating the effect of FDI on DI in the food manufacturing sector for developing, economies in transition and developed countries.

**Methods: **Using an unbalanced panel data of 49 countries from 1993 to 2016, from FAOSTAT, estimated by the system generalised method of moments (GMM), the Wald statistics for the short and long-run effects of FDI on DI were computed for the development groups.

**Results:** Developed economies experienced a crowd-out effect of FDI on DI in the short run, whilst the others experienced no significant effect. In the case of the long run, the food manufacturing sectors of all three development groups exhibited a crowd-out effect. The effect in the long run for all development groups together is a crowd-in. Analysing all country groups together could mask the results of the various country groups.

**Conclusions:** A review of investment policies to prioritise FDI entry mode that favour domestic investment is needed. Improvement of the investment regulatory and administrative efficiency among others are recommended.

## Introduction

In food manufacturing, relatively bulky, perishable and typically inedible raw materials are converted into more useful, shelf-stable and palatable foods or potable beverages with the aid of various unit operations and technologies ( FAO, 2010; Stadler *et al.,* 2020). Food processing contributes to food security by minimising waste and loss in the food chain and by increasing food availability and marketability. Food manufacturing also improves food quality and safety ( FAO, 2010; Leonard *et al.,* 2020; Phan *et al.,* 2020; Stadler *et al.,* 2020).

Global gross domestic product (GDP) of food manufacturing for 2017 is estimated at US$1.68 trillion ( FAOSTAT, 2020). The sector appears to be relatively more important to developing and economies in transition as it constitutes 3.03% and 3.11% respectively of GDP than developed countries (2.10%) ( FAOSTAT, 2020) ^1^. As the sector needs more investments for growth and development ( Hine, 2015; Primanthi, 2015), many countries have pursued policies to attract foreign direct investment (FDI) into the food manufacturing sectors of their respective economies to reap benefits including investment accumulation, technology transfer and job creation ( de Mello Jr, 1997; Farla *et al.,* 2016; Kosova, 2010). Aside from these benefits, there is evidence that the inflow of FDI compliments domestic investment (DI) ( Farla *et al.,* 2016; Mileva, 2008) or substitute DI ( Budang & Hakim, 2020; Oualy, 2019) for whole economy cases as well as the agricultural sector ( Djokoto *et al.,* 2014). Agosin & Machado (2005) and Gallova (2011) have reported a neutral effect of FDI on DI. A complementary effect of FDI on DI is desirable as an increase in FDI promotes further domestic investment. In the case of the substitution effect, increased FDI decreases DI, which could be detrimental. In the case of the neutral effect, beyond the economic benefits stated earlier, there are no additional benefits related to DI. In light of the foregoing, what is the evidence of FDI on DI for the food manufacturing sector of different country groups?

Pagoulatos (1983) studied the effect of FDI in US food and tobacco manufacturing on domestic economic performance. Djokoto *et al.* (2014) investigated the effect of FDI on the DI of agriculture in Ghana. In cross-country studies, Agosin & Machado (2005) studied the effect of FDI on DI for whole economies of developing countries, Budang & Hakim (2020) for Asian countries, Gallova (2011) and Mileva (2008) on transition economies, Pilbeam & Oboleviciute (2012) for EU countries, and Wang (2010) for developed and developing countries. Whilst the cross-country studies focused on whole economies, these were limited to transition, developing, or developed and developing countries. The single-country studies failed to address the effect of FDI on DI for food manufacturing. This paper fills these gaps by investigating the effect of FDI on DI in the food manufacturing sector for developing, economies in transition and developed countries.

The implications of the effects of FDI inflows on DI are issues that require increased attention by international economists and the facet of food manufacturing concerns agricultural economists as well. Analysing this topic requires linking development and investment theories within the food manufacturing sector. Understanding the issues surrounding the effect of FDI on DI in the food manufacturing sector across countries at varying levels of development is essential in understanding short- and long-term adjustments facing investors and economic managers as food systems become more integrated into the global economy.

In what follows, the existing theories and empirical evidence regarding the effect of FDI on DI are presented. The section on results and discussions is preceded by the outline of the data, modelling and estimation procedure. The conclusions and recommendations complete the paper.

### Relevant theories

In line with the key issues in the paper, the theoretical review is constituted into four parts. The first, theories of development seek to explain the differences in the level of development of countries, one of the foci of the paper. Space is provided for FDI theories as these explain the role of the key variable that influences DI. DI is the dependent variable and thus deserves space, hence the investment theories. To tie in, in the interaction of FDI and DI, the theoretical explanation of the link between FDI and DI is outlined as well.

Many theories have been propounded regarding economic development. These have been classified differently. The classification used here is modernisation, dependency, world systems and globalisation. Modernisation theory uses a systematic process to move underdeveloped countries to a more sophisticated level of development ( Reyes, 2001). This theory which stresses the importance of political development in the progress and climactic improvement of a nation’s economic standing, also acknowledges social and cultural reforms. Further, it seeks to explain inequality within or between states by identifying different values, systems and ideas held by different nations ( Martinussen, 1997). Also, it gives attention to the shift of modern technology and development institutions and labour habits complementary to industrial production ( Chase-Dunn, 1975).

Dependency theory seeks to improve modernisation theory, combines elements from a neo-Marxist theory and adopts a revolution of underdeveloped nations model ( Reyes, 2001). This, together with the Marxist position seeks to explain the origin of surpluses, the basis of theoretical evaluation of progress, and inequality. The divide between developed and under-developed countries is well established. The reason for the difference between the divide and what interventions are required to narrow the divide engages the attention of this theory. ( Haque, 1999; Muuka, 1997; Shen & Williamson, 2001; Todaro, 2000).

The "World System" is multiple cultural systems with a single division of labour. The basic feature of this system is having a pool of labour in which different divisions and areas are dependent upon each other in exchanging the provisions of those areas ( Wallerstein, 1974). In using other levels of quantitative analysis, this theory argues that international trade specialisation and transfer of resources from less developed countries to developed countries prevents development in less developed countries by making them rely on core countries and by encouraging peripheralisation ( Szymanskiv, 1982). The theory views the world economy as an international hierarchy of unequal relations. Through the world system, a country can change its position in the global hierarchy ( Szymanskiv, 1982).

Globalisation as a theory of development uses a world mechanism of greater integration with emphasis on the sphere of economic transactions ( Reyes, 2001). Whether as an event of interdependence throughout different countries of the world in different aspects of communication, trade, and finance or continuous and widespread unification among different countries, it focuses on communications and international ties directed at cultural and economic factors in communication systems. This theory explains inequality by identifying cultural and economic factors in global connection ( Zineldin, 2002). Globalisation endangers global competition. Whilst this could hurt especially developing countries, firms in developing countries can respond by increasing production and efficiency thereby improving their economic situation. Also, rationalisation of production on a global scale and the spread of technology result from globalisation ( Intriligator, 2004; Stiglitz, 2004a; Stiglitz, 2004b; Tanzi, 2004).

Foreign direct investment theories can be viewed from three perspectives. The first perspective, which is internationalisation theory, explains why firms often prefer FDI to license as a strategy for entering a foreign market ( Hymer, 1976). In this theory, FDI is preferred for licensing and exporting. Licensing may result in a firm giving away valuable technological expertise to a potential foreign investor at a fee. This does not give a firm control over manufacturing, marketing, and strategy in a foreign country that may be required to maximise its profitability. Unfortunately, the competitive advantages of management, marketing, and manufacturing capabilities are not amenable to licensing.

The second perspective relates to the patterns of FDI. In oligopolistic industries, firms invest in other countries as a strategy by following their domestic competitors ( Knickerbocker, 1973). Related to this is the product life cycle hypothesis ( Vernon, 1966). Firms invest in other advanced countries when local demand in those countries grows large enough to support local production. Production is subsequently shifted to developing countries when product standardisation and market saturation give rise to price competition and cost pressures.

The third perspective is the Dunnings’ eclectic paradigm. Dunning (1977); Dunning (1988); Dunning (2001) stated that the extent, geography, and industrial composition of foreign production undertaken by the multinational enterprise is determined by the interaction of three sets of interdependent variables which, themselves, comprise the components of three sub-units, namely; ownership, location and internationalisation (OLI). All other factors unchanged, the greater the competitive advantages of the investing firms, relative to those of other firms, the more they are likely to be able to engage in or increase, their foreign production. This is the own competitive advantage. For the location, the more the immobile, natural or created endowments, needed by the firms to use jointly with their competitive advantages, favour a presence in a foreign rather than a domestic location, the more firms will choose to supplement or take advantage of their own specific advantages by engaging in FDI. The multinational enterprise, thus, would undertake activities to add value to its operations. Internalisation, the final competitive advantage, offers a framework for evaluating alternative ways in which firms may organise the creation and exploitation of their core competencies. These range from buying and selling goods and services in the open market, through a variety of inter-firm non-equity agreements, to the integration of intermediate product markets and outright purchase of a foreign corporation.

The many theories of investment include accelerator theory, cash flow theory, neoclassical theory, modified neoclassical theory and Q theory. Chenery (1952); Clark (1917) and Koyck (1954) among others, emphasised the accelerator model that sought to explain business cycles. Eisner & Nadiri (1968); Eisner & Strotz (1963) incorporated profit and investment equation into the accelerator theory.

Cashflow, the resources left after paying dividends to shareholders determines investment from internal sources. Past profits drive future investment decisions ( Kuh, 1963; Meyer & Kuh, 1955; Strong & Meyer, 1990). The decision to invest is informed by the cost of capital which in turn is driven by the profit maximising behaviour of the firm ( Jorgenson, 1963; Jorgenson, 1971; Modigliani & Miller, 1965). Biscoff (1971) extended the neoclassical theory to show that it is possible to alter the capital-output ratio so that later, the substitution effect of output goes to zero. Consequently, the investment can be effectively induced by changes in the capital-output ratio.

The Q theory of investment notes that, if investors seek to maximise the market value of the firm, they will add to their capital stock whenever the marginal addition to the firm’s market value exceeds the replacement cost of the capital stock ( Brainard & Tobin, 1968; Tobin, 1969). This theory is driven by the ratio of the market value of capital to its replacement cost, unlike the neoclassical theory which is driven by the cost of capital.

The fourth theoretical perspective is the interaction between FDI and DI. Markusen & Venables (1999) theorised the relationship between multinational enterprises (MNEs) and domestic firms on the entry of MNEs to be a competition effect and a linkage effect. Regarding the competition effect, the entry of MNEs increases competition in the final product industry and reduces the profitability of domestic firms in the same industry. Consequently, domestic firms do exit the market. For the linkage effect, the entry of multinational corporations (MNCs) could cause the demand for domestic production of intermediate inputs to rise. This tends to create an increased number of domestic firms in the intermediate inputs industry. Barro & Sala-i-Martin (2004) have also acknowledged these effects.

Barrios *et al.* (2005) posit that as the number of MNEs increases in the host country, the number of domestic firms might drop first and then rise. Assuming that MNEs enter the downstream (final product) market, an increase in the total number of firms (both foreign and domestic) in the downstream industry will decrease the price level for the final product. The possibility is the reduction of profit for all firms and thereby forcing some domestic firms to exit. This would happen if the MNEs are more productive than domestic firms. On the other hand, the possible linkage effect can increase the number of upstream domestic firms and then reduce the cost of production in the downstream industry for both MNEs and domestic firms. Therefore, the number of domestic firms in the host country will eventually go up.

### Previous empirical studies

The effect of FDI on DI could be complementary, substitutional or neutral. In the developing country context, Agosin & Machado (2005); Ahmad *et al.* (2018); Djokoto (2013); Josue *et al.* (2014); Oualy (2019) and Wang (2010) have reported the neutral effect of FDI on DI in the short run. Kim & Seo (2003) found a significant crowd-in effect of FDI on DI for the whole of Korea. In the long run, whilst Agosin & Machado (2005); Morrissey & Udomkerdmongkol (2012) and Mutenyo & Asmah (2010) reported crowding out effect of FDI on DI for Latin America, developing countries and sub-Saharan Africa respectively, Djokoto *et al.* (2014) and Farla *et al.* (2016) found the crowding-in effect of FDI on DI for Ghanaian agriculture and developing countries respectively. Babu (2021) found a crowding-out effect for India based on data from 1990/91 to 2014/15. Djokoto *et al.* (2014) attributed the positive effect to technology diffusion and spillover of management know-how by MNEs and vertical inter-firm linkages with domestic firms.

Mileva (2008) found a crowding-in effect of FDI on DI for economies in transition using data from 1995–2005 in the short run and long run, despite the presence of less developed financial markets and weaker institutions. Cooperation instead of competition can be adduced for the crowd-in effect. Jude (2019) and Kejžar (2016) reported switching effects of FDI on DI for transition economies; short-run crowding-out effect to long-run crowding-in effect. The magnitude of the possible linkage and spillover effects over time overcomes the initial competition effect. Also, the period for the long run is such as would allow completed plants to run for some time to generate output and associated benefits. Technology, knowledge transfer, employment and expenditure on social responsibility would come to fruition.

Gallova (2011) reported that FDI exerted no effect on DI for Bulgaria and Romania but crowding in effect for Croatia and Slovenia. For the Balkans, for the period 1993–2009, the effect was crowding out. Gallova (2011) explained that foreign companies do employ the services of the same suppliers as their parent companies that are not in the host country. MNEs tend to also bring with them to the host country, foreign producers from which they take the components necessary for their production. Additionally, foreign-owned companies are so strong in individual sectors and effectively functioning. When domestic firms fail to assert themselves effectively and establish cooperation with MNEs they tend to be crowded out of the market. In the long run, however, based on the panel data from 1993–2009, Gallova (2011) found no long-run effect for the Balkans and the individual countries. For the Czech Republic and Hungary, Mišun & Tomšík (2002) concluded on the long run crowding in effect but the crowding-out effect for Poland. Other studies on developed countries also concluded with mixed outcomes regarding FDI and DI. Pilbeam & Oboleviciute (2012) found a short and long-run crowd-out effect for EU-15 (Austria, Belgium, Denmark, Finland, France, Germany, Greece, Ireland, Italy, Luxembourg, Netherlands, Portugal, Spain, Sweden and the United Kingdom) but no effect for EU-12 (Bulgaria, Czech Republic, Estonia, Cyprus, Latvia, Lithuania, Hungary, Malta, Poland, Romania, Slovenia, and Slovakia). In the work of Polat (2017), the effect of FDI on DI was neutral for Organisation for Economic Co-operation and Development (OECD) countries for data covering 2006 to 2013, just as Wang (2010) for developed countries in the long run. In the short run, however, Wang (2010) found the crowding-out effect for developed countries.

From the empirical review, some studies dwelled on whole economies across countries whilst others covered one or single economy. One single economy study addressed agriculture. Food manufacturing, the offtaker of agricultural production and the product supplier for marketing in the agribusiness value chain, has not been given attention. This paper focuses on this. 

## Methods

### Data

Some previous studies used time-series data ( Chen *et al.,* 2017; Djokoto, 2013; Djokoto *et al.,* 2014; Gallova, 2011; Kim & Seo, 2003) whilst others employed panel data ( Agosin & Machado, 2005; Ahmad *et al.,* 2018; Budang & Hakim, 2020). This study used panel data. The data consist of 49 countries for the period 1993 to 2016 ( Table 1). The description of the data and measurement are outlined in Table 2. All the data was obtained from FAOSTAT (2020). The panel structure enables the pooling of the observations for each unit over time resulting in more observations and smaller standard errors. Further, the data structure allows for analysing the dynamic behaviour of units and dynamic models; using lagged outcome variables as explanatory variables ( Greene, 2003; Gujarati & Porter, 1999; Wooldridge, 2002).

**Table 1.** List of countries in the study.

Developing		Developed		
Brazil	Trinidad and Tobago	Austria	Greece	Portugal
Costa Rica	Tunisia	Belgium	Hungary	Romania
Fiji	Turkey	Bulgaria	Iceland	Slovakia
India		Croatia	Ireland	Slovenia
Israel	**Transition**	Cyprus	Italy	Spain
Malawi	Armenia	Czechia	Japan	Sweden
Mexico	Kazakhstan	Denmark	Latvia	UK
Philippines	Russian Federation	Estonia	Lithuania	USA
Republic of Korea		Finland	Luxembourg	
Singapore		France	Netherlands	
Thailand		Germany	Poland	

Note: The development designations are based on United Nations (2020). World Economic Situation and Prospects, Statistical annexe

**Table 2.** Variable descriptions.

Variable	Definition	Measurement
*DI*	Domestic investment into food manufacturing	Gross fixed capital formation of food manufacturing divided by food manufacturing value-added both in current US$
*DI_1*	Domestic investment into food manufacturing with a one-year lag	Gross fixed capital formation of food manufacturing divided by food manufacturing value-added with a one-year lag both in current US$
*DI_2*	Domestic investment into food manufacturing with a two-year lag	Gross fixed capital formation of food manufacturing divided by food manufacturing value-added with a two-year lag both in current US$
*FDI*	Foreign direct investment into food manufacturing	Foreign direct investment into food manufacturing is divided by food manufacturing value-added both in current US$
*FDI_1*	Foreign direct investment into food manufacturing with a one-year lag	Foreign direct investment into food manufacturing is divided by food manufacturing value-added with a one-year lag both in current US$
*FDI_2*	Foreign direct investment into food manufacturing with a two-year lag	Foreign direct investment into food manufacturing is divided by food manufacturing value-added with a two-year lag both in current US$
*GR_1*	The growth rate of food manufacturing with a one-year lag	The growth rate of food manufacturing value-added at 2010 prices with a one-year lag
*GR_2*	The growth rate of food manufacturing with a two-year lag	The growth rate of food manufacturing value-added at 2010 prices with a two-year lag
*DVP*	Developing economies	Developing countries=1, and 0 otherwise
*TRS*	Transition economies	Transition economies=1 and 0 otherwise
*DVD	Developed economies	Developed economies = 0, the reference.
*FDI_DVP*	Interaction of FDI and DVP	Value = FDI and 0 otherwise
*FDI_DVP_1*	Interaction of FDI_1 and DVP	Value = FDI_1 and 0 otherwise
*FDI_DVP_2*	Interaction of FDI_2 and DVP	Value = FDI_2 and 0 otherwise
*DI_DVP_1*	Interaction of DI_1 and DVP	Value = DI_1 and 0 otherwise
*DI_DVP_2*	Interaction of DI_2 and DVP	Value = DI_2 and 0 otherwise
*FDI_TRS*	Interaction of FDI and TRS	Value = FDI and 0 otherwise
*FDI_TRS_1*	Interaction of FDI_1 and TRS	Value = FDI_1 and 0 otherwise
*FDI_TRS_2*	Interaction of FDI_2 and TRS	Value = FDI_2 and 0 otherwise
*DI_TRS_1*	Interaction of DI_1 and TRS	Value = DI_1 and 0 otherwise
*DI_TRS_2*	Interaction of DI_2 and TRS	Value = DI_2 and 0 otherwise
*FDI_DVD_1	Interaction of FDI_1 and DVD	Value = FDI_1, the reference
*DI_DVD_1	Interaction of DI_1 and DVD	Value = DI_1, the reference

Note: 1. All data from FAOSTAT: http://www.fao.org/faostat/en/#home. The development designations are based on United Nations (2020). 2. * These variables went into Equation 2 as latent variables (reference).

### Modelling

Agosin & Machado (2005) developed a theoretical model based on a neoclassical investment model using partial adjustment of capital stock and adaptive expectations of economic growth in which FDI has been included. This model has been applied in the study of the relationship between FDI and DI ( *See for example*, Budang & Hakim, 2020; Oulay, 2019; Pilbeam & Oboleviciute, 2012). The Agosin & Machado (2005) model is adapted and specified in Equation 1.

$$\begin{array}{l} DI_{i,t}=\beta_{0}+\beta_{1}\;DI_{i,t-1}+\beta_{2}\;DI_{i,t-2}+\beta_{3}\;FDI_{i,t}+\beta_{4}\;FDI_{i,t-1}+\beta_{5}\;FDI_{i,t-2}+\beta_{6}\;GR_{i,t-1}+\\ \beta_{7}\;GR_{i,t-2}+\beta_{8}\;TIME_{i,t}+\varepsilon_{i,t} \end{array}\;1$$

Where *ε _I,t_* is the error term.

To account for the levels of development of the countries in the data, for which reason the effects of FDI on DI may differ, Equation 1 is re-specified:

$$\begin{array}{l} DI_{i,t}=\beta_{0}+\beta_{1}\;DI_{i,t-1}+\beta_{2}\;DI_{i,t-2}+\beta_{3}\;FDI_{i,t}+\beta_{4}\;FDI_{i,t-1}+\beta_{5}\;FDI_{i,t-2}+\beta_{6}\;GR_{i,t-1}+\;\beta_{7}\;GR_{i,t-2}\\ \;+\;\beta_{8}\;FDI\_DVP_{i,t}+\beta_{9}\;FDI\_DVP_{i,t-1}+\beta_{10}\;FDI\_DVP_{i,t-2}+\beta_{11}\;DI\_DVP_{i,t-1}\\ \;+\;\beta_{12}\;DI\_DVP_{i,t-2}+\beta_{13}\;FDI\_TRS_{i,t}+\beta_{14}\;FDI\_TRS_{i,t-1}+\beta_{15}\;FDI\_TRS_{i,t-2}\\ \;+\beta_{16}\;DI\_TRS_{i,t-1}+\beta_{17}\;DI\_TRS_{i,t-2}+\beta_{18}\;TIME_{i,t}\\ \;+\gamma_{i,t} \end{array}\;2$$

All the variables and their sources are described in Table 2. The classification of countries based on the level of development reflects the basic conditions ( United Nations, 2020). It does indirectly capture some macroeconomic variables that could otherwise have been included as control variables. 

Let *β _STk_* be the short-run effects, where *k*=1,2, 3,4 are developing countries, economies in transition, developed countries and all development groups, respectively. Then,

$$\;{\hat{\beta }}_{ST1}=\beta_{3}+\beta_{8}=0\;3$$

for developing countries.

In the case of transition economies,

$$\;{\hat{\beta }}_{ST2}=\beta_{3}+\beta_{13}=0\;4$$

Whilst for developed countries,

$$\;{\hat{\beta }}_{ST3}=\beta_{3}=0\;5$$

For the three-country group effects, the null hypothesis for the short run is

$$\;{\hat{\beta }}_{ST4}=\beta_{3}+\beta_{8}+\beta_{13}=0\;6$$

Failure to reject these null hypotheses with a chi-square test implies FDI has no short-run or contemporaneous effect on DI. Rejection of the null hypothesis and that *β _STk_* > 0 means a contemporaneous crowd-in effect of FDI on DI. Alternatively, *β _STk_* < 0 means a contemporaneous crowd-out effect of FDI on DI. 

The long-run effect is represented by ${\hat{\beta }}_{LT1},$ where *k*=1,2,3,4 as previously. Then, the null hypotheses for the long-run effects ^2^ are

$$\;{\hat{\beta }}_{LT1}=\sum_{j=1}^{5}{\hat{\beta }}_{j}+\;\sum_{j=8}^{12}{\hat{\beta }}_{j}=1\;7$$

for developing countries. For transition economies

$$\;{\hat{\beta }}_{LT2}=\sum_{j=1}^{5}{\hat{\beta }}_{j}+\;\sum_{j=13}^{17}{\hat{\beta }}_{j}=1\;8$$

In the case of developed countries,

$$\;{\hat{\beta }}_{LT3}=\sum_{j=1}^{5}{\hat{\beta }}_{j}=1\;9$$

For all development groups, in the long run,

$$\;{\hat{\beta }}_{LT4}=\sum_{j=1}^{5}{\hat{\beta }}_{j}+\;\sum_{j=8}^{17}{\hat{\beta }}_{j}=0\;10$$

a. With a chi-squared test, if it is not possible to reject the null hypothesis that ${\hat{\beta }}_{LT1}=1,$ then, in the long run, an increase in FDI of one currency unit, becomes one currency unit of additional total investment. Stated differently, investment by MNEs simply matches one-to-one to investment by domestic firms, and no macroeconomic externalities are stemming from FDI.

b. If the null hypothesis, ${\hat{\beta }}_{LTK}$ = 1 is rejected and ${\hat{\beta }}_{LTK}$ > 1, this is evidence of the long-run crowd-in effect of FDI by DI. Thus, in the long run, one additional currency unit of FDI becomes more than one additional currency unit of total investment.

c. Now, consider the case that the null hypothesis, ${\hat{\beta }}_{LTK}$ = 1 is rejected and ${\hat{\beta }}_{LTK}$ < 1, there is then a long-run crowd-out effect. One additional currency unit of FDI leads to less than one currency unit increase in total investment. This means there is a displacement of DI by FDI.

### Estimation procedure

The pertinent literature used the ordinary least squares (OLS) estimator ( Chen *et al.*, 2017; Djokoto, 2013; Djokoto *et al.,* 2014; Gallova, 2011; Kim & Seo, 2003). Agosin & Machado (2005); Pilbeam & Oboleviciute (2012) and Polat (2017) used a generalised method of moments (GMM). GMM has some weaknesses. First, in the presence of the lagged dependent variables on the right-hand side of the equation and the time-invariant country-specific factors, the fixed-effects estimator would yield inconsistent estimates because of the correlation between the lagged dependent variable and the error terms. Taking the first differences to remove the time-invariant country-specific factors still results in a correlation between the error term and the DI. Second, FDI inflows are likely to be endogenous and determined jointly with the DI. Impliedly, there is a two-way relationship between DI and FDI ( Al-sadiq, 2013; Arellano & Bond, 1991; Arellano & Bover, 1995). The system-GMM estimator enables controlling for the unobserved country-specific factors and solves the correlation problem by using a set of internal instruments based on the assumption of no second-order serial correlation in the first-differenced idiosyncratic errors, and the independent variables are weakly exogenous. In this way, the estimated coefficients would then not be subject to bias from an omitted variable. Using a series of internal instrumental variables based on lagged values of the dependent and independent variables, the system-GMM estimator solves the endogeneity problem as well. All these are accomplished by combining in one system, the regression in differences with the regression in levels under the assumption that there is no correlation between the differences of the right-hand side variables and the unobserved country-specific effects ( Al-sadiq, 2013; Arellano & Bond, 1991; Arellano & Bover, 1995; Blundell & Bond, 1998). The assumption of no serial correlation is testable. Whilst first-order autocorrelation (AR(1)) of the errors is permissible, that of the second-order (AR(2)) is inadmissible. As internal instruments are used, the Sargan test tests whether the overidentifying restrictions are valid. Rejection of the null hypothesis would indicate that the instruments are not valid, therefore, the estimates are not reliable. 

## Results and discussion

### Summaries

Due to the unbalanced nature of the panel, the number of observations for the variables is not the same. The observations ranged from 611 to 1,344. The growth rate (GR) for food manufacturing value-added averaged about 3% ( Table 3). The means of DI_1 for developing, transition and developed economies were respectively, 0.0369, 0.0079 and 0.1083. These are statistically distinguished ( Table 4). Thus, developed countries on average accumulate about 11% of food manufacturing domestic investment as GDP whilst developing countries manage about 4%. Economies in transition posted less than 1%. The positive value of FDI implies that there has been a transfer of FDI from one economy to the other. This is symptomatic of international capital transfer ( Dunning, 1977; Dunning, 1988; Dunning, 2001; Reyes, 2001). The means of FDI_1 of the development groups is likewise statistically distinguished. However, the values constitute less than 1% of food manufacturing value-added. As in the case of DI, developed countries post a higher value of 0.99%. Transition economies, however, seem to attract more FDI dollars of food manufacturing GDP than developing economies. 

**Table 3.** Summary statistics.

Variable	Observations	Mean	Standard deviation	Minimum	Maximum
*Country ID*	1,344	28.5	16.1693	1	56
*Year ID*	1,344	12.5	6.9248	1	24
*DI*	1,018	0.2050	0.1292	0	0.9940
*DI_1*	1,009	0.2040	0.1283	0	0.9630
*DI_2*	995	0.2046	0.1311	0	0.9630
*FDI*	639	0.0326	0.1740	-3.2364	0.8835
*FDI_1*	626	0.0327	0.1747	-3.2364	0.8835
*FDI_2*	611	0.0316	0.1740	-3.2364	0.8835
*GR_1*	984	0.0289	0.0986	-0.5586	0.7298
*GR_2*	972	0.0305	0.0983	-0.5586	0.7298
*DVP*	1,344	0.3214	0.4672	0	1
*TRS*	1,344	0.0536	0.2253	0	1
*DVD*	1,320	0.6364	0.4812	0	1
*DI_DVP_1*	1,344	0.0369	0.0909	0	0.5393
*DI_TRS_1*	1,344	0.0079	0.0429	0	0.4553
*DI_DVD_1*	1,344	0.1083	0.1426	0	0.9630
*FDI_DVP_1*	1,344	0.0023	0.0179	-0.0911	0.4064
*FDI_TRS_1*	1,344	0.0030	0.0389	-0.2459	0.6773
*FDI_DVD_1*	1,344	0.0099	0.1129	-3.2364	0.8835
*DI_DVP_2*	1,344	0.0375	0.0940	0	0.6001
*DI_TRS_2*	1,344	0.0079	0.0429	0	0.4553
*FDI_DVP*	1,344	0.0023	0.0180	-0.0911	0.4064
*FDI_DVP_2*	1,344	0.0024	0.0177	-0.0911	0.4064
*FDI_TRS*	1,344	0.0029	0.0392	-0.2459	0.6773
*FDI_TRS_2*	1,344	0.0031	0.0390	-0.2459	0.6773

**Table 4.** F test and pairwise correlations.

F–test				
Groups	Mean	Variance	F value	
DI_DVP_1	0.0369	0.0083	353.5502***	
DI_TRS_1	0.0079	0.0018		
DI_DVD_1	0.1083	0.0203		
FDI_DVP_1	0.0023	0.0003	4.7991***	
FDI_TRS_1	0.0030	0.0015		
FDI_DVD_1	0.0099	0.0128		
Correlation–short run				
	DI_DVP	DI_TRS	DI_DVD	DI
FDI_DVP	0.1991***			
FDI_TRS		0.4302***		
FDI_DVD			0.0917*	
FDI				0.0437
Correlation–long run				
	DI_DVP_1	DI_TRS_1	DI_DVD_1	DI
FDI_DVP_1	0.2034***			
FDI_TRS_1		0.4568***		
FDI_DVD_1			0.0895***	
FDI_1				0.0318

As the key variables are FDI and DI, linear correlation coefficients were computed (second panel of Table 4). There is a significant positive correlation between FDI and DI for each developing country group. For the country groups together, however, the positive relationship is not statistically distinguishable from zero. Similarly, in the long run, there are positive significant relationships between FDI and DI but no significant relationship for all country groups. Although the linear correlation coefficients could give an early indication of the effect between the variables, the influence of other variables during the estimation process could change the effect shown by the linear correlation coefficient.

### Sensitivity analysis

Up to 15 lags were estimated for the system GMM. This is because the lag length is known to influence the size of estimates especially, coefficients and standard errors, which are crucial in the calculation of the short and long-run effects of FDI on DI. Based on the number of significant coefficients and the level of significance lag 4 was selected. The selected system GMM results are reported in Table 5 as model 4.

**Table 5.** Results of sensitivity analysis of the estimations for the level of development and FDI on DI.

	1	2	3	4	5
	Only key variables	1 with GRs only	1 with TIME only	1 with GRs and TIME	4 without ‘outliers’
VARIABLES	DI	DI	DI	DI	DI
*DI_1*	0.7392*** (0.0291)	0.7463*** (0.0277)	0.7229*** (0.0270)	0.7078*** (0.0338)	0.6495*** (0.0424)
*DI_2*	0.0324 (0.0286)	-0.0004 (0.0284)	0.0172 (0.0218)	0.0042 (0.0353)	0.0408 (0.0249)
*FDI*	-0.0156*** (0.0040)	-0.0180*** (0.0046)	-0.0159*** (0.0035)	-0.0154*** (0.0053)	-0.0138*** (0.0052)
*FDI_1*	-0.0188*** (0.0034)	-0.0206*** (0.0052)	-0.0155*** (0.0036)	-0.0195*** (0.0059)	-0.0181*** (0.0053)
*FDI_2*	-0.0101** (0.0047)	-0.0119* (0.0071)	-0.0081* (0.0045)	-0.0108* (0.0063)	-0.0104 (0.0064)
*GR_1*	-	0.0190* (0.0098)	-	0.0221** (0.0102)	0.0154 (0.0128)
*GR_2*	-	0.0251*** (0.0085)	-	0.0261*** (0.0096)	0.0247** (0.0102)
*FDI_DVP*	0.0686 (0.0502)	0.0416 (0.0442)	0.0567 (0.0496)	0.0211 (0.0617)	0.0099 (0.0574)
*FDI_DVP_1*	-0.0141 (0.0296)	-0.0300 (0.0385)	-0.0429 (0.0424)	-0.0097 (0.0469)	-0.0345 (0.0560)
*FDI_DVP_2*	0.0050 (0.0724)	-0.0176 (0.0614)	-0.0754 (0.0985)	-0.0353 (0.0905)	-0.0226 (0.1480)
*DI_DVP_1*	-0.3209*** (0.0614)	-0.3858*** (0.0841)	-0.3560*** (0.0667)	-0.3066*** (0.0667)	-0.3291*** (0.0824)
*DI_DVP_2*	-0.0737 (0.0550)	0.0526 (0.0727)	-0.0112 (0.0836)	0.0354 (0.1058)	0.0179 (0.0946)
*FDI_TRS*	0.0710*** (0.0247)	0.0270 (0.0334)	0.0593*** (0.0225)	0.0620* (0.0345)	0.0414 (0.0300)
*FDI_TRS_1*	0.0476* (0.0248)	0.0021 (0.0332)	0.0410 (0.0253)	0.0445 (0.0351)	0.0211 (0.0333)
*FDI_TRS_2*	0.0476 (0.0470)	-0.0347 (0.0650)	0.0205 (0.0427)	0.0319 (0.0678)	0.0111 (0.0560)
*DI_TRS_1*	0.4506*** (0.1290)	0.4146*** (0.1213)	0.2711** (0.1294)	0.3814** (0.1577)	0.5224*** (0.1955)
*DI_TRS_2*	-0.5841*** (0.1429)	-0.4055*** (0.1285)	-0.3903*** (0.1217)	-0.5416*** (0.1793)	-0.6297*** (0.1820)
*TIME2*	-	-	0.0010*** (0.0003)	0.0009** (0.0004)	0.0005 (0.0004)
Constant	0.0653*** (0.0054)	0.0699*** (0.0065)	0.0614*** (0.0085)	0.0653*** (0.0091)	0.0739*** (0.0064)
Model properties					
Observations	471	460	471	460	414
Number of countries	46	45	46	45	41

To establish the consistency of the estimates of model 4, four other models; 1–3 and 5, were estimated. Model 1 includes only the key variables; the lags of DI and the FDI and its lags. In model 2, GRs are added to model 1. In model 3, the GRs are dropped, and the TIME variables are added. Model 5 includes all variables as in model 4, but without some observations. Data of large economies could influence the overall results. Thus, for developed countries, the observations for India and Brazil were dropped. For economies in transition, Russia dropped whilst the United States of America dropped from the observations of developed economies ( Table 1). The only departures are FDI_TRS1 for model 1, FDI_TRS for model 2, and FDI_2 and FRI_TRS for model 5 ( Table 5). For model 1, the coefficient of the variables is weakly significant at 10% whilst those for all other models are statistically insignificant. For model 2, the coefficient of the variable is statistically insignificant together with that of model 5 but the coefficients of the variable for all models are statistically significant at either 1% or 10%. For model 5, the coefficient for FDI_2 is statistically insignificant, whilst one of the lag 2 of FDI is statistically significant at 5% whilst the other three are significant at 10%. It is instructive to note that, despite these minor departures, the size and sign of the magnitudes of the coefficients are comparable. Thus, generally, the estimates are not sensitive to the changes in variables and observations and are consistent and robust. 

As the goal of the estimations is to ascertain the short and long-run effects of FDI on DI, the estimates of models 1 – 5, were used to compute the effects ( Table 6). The short and long-run effects are consistent across all five models for developing and developed economies and for the long run for economies in transition. The only departures are for the short run in the case of transition economies; statistically significant effects for model 1 and model 3. This is not surprising. Transition economies in the data are made up of three countries; Armenia, Kazakhstan and the Russian Federation ( Table 1). Thus, the results of the group are more likely to be responsive to the deletion and inclusion of variables and observations than those country groups with larger numbers. Combining all the country groups, the short-run effects for model 1 differ from those of other models. Also, the statistics of the long-run effect of model 5 are statistically insignificant, unlike others that are statistically significant, although the magnitude is like others. Model 5 has two coefficients that were statistically insignificant more than the other models, suggesting high standard errors. This certainly contributed to the statistically insignificant Wald statistic. Overall, however, the results of the short and long-run effects are generally consistent and robust to the estimations of model 4, the standard. 

**Table 6.** Results of the sensitivity of short- and long-run effects to changes in variables and observations.

	1	2	3	4	5
	Key variables only	Plus GRs only	Plus TIME only	Plus GRs and TIME	Without ‘outliers’
Developing economies					
Short run	0.0530	0.0235	0.0408	0.0056	-0.0040
Long run	0.3920***	0.3561***	0.2719***	0.3711***	0.2894**
Economies in transition					
Short run	0.0554***	0.0090	0.0433***	0.0466	0.0276
Long run	0.7598***	0.6988***	0.7022***	0.6443***	0.6141***
Developed economies					
Short run	-0.0156***	-0.0180***	-0.0159***	-0.0154***	-0.0138***
Long run	0.7271***	0.6953***	0.7006***	0.6662***	0.6479***
Combined					
Short run	0.1240***	0.0505	0.0682	0.0676	0.0098
Long run	1.9678***	1.8512***	1.6747***	1.7731***	1.5574

## Discussion of estimations

The AR(1) (Autoregressive 1) and AR(2) (Autoregressive 2) statistics fall within expectations, with first-order serial correlation with no second-order serial correlation ( Table 7). The Sargan statistics are also statistically insignificant. That is the failure to reject the null hypothesis that the instruments are not valid. Impliedly, the estimates are reliable.

**Table 7.** Results of the main model, model 4.

	System GMM
Variables	DI
*DI_1*	0.7078 ^***^ (0.0338)
*DI_2*	0.0042 (0.0353)
*FDI*	-0.0154 ^***^ (0.0053)
*FDI_1*	-0.0195 ^***^ (0.0059)
*FDI_2*	-0.0108 ^*^ (0.0063)
*GR_1*	0.0221 ^**^ (0.0102)
*GR_2*	0.0261 ^***^ (0.0096)
*FDI_DVP*	0.0211 (0.0617)
*FDI_DVP_1*	-0.0097 (0.0469)
*FDI_DVP_2*	-0.0353 (0.0905)
*DI_DVP_1*	-0.3066 ^***^ (0.0667)
*DI_DVP_2*	0.0354 (0.1058)
*FDI_TRS*	0.0620 ^*^ (0.0345)
*FDI_TRS_1*	0.0445 (0.0351)
*FDI_TRS_2*	0.0319 (0.0678)
*DI_TRS_1*	0.3814 ^**^ (0.1577)
*DI_TRS_2*	-0.5416 ^***^ (0.1793)
*TIME*	0.0009 ^**^ (0.0004)
Constant	0.0653 ^***^ (0.0091)
Observations	460
Number of countries	45
Number of instruments	120
Wald test	25,947 ^***^
AR(1)	-2.7588 ^***^
AR(2)	-1.0174
Sargan statistic	37.3165

Note: 1. Standard errors in parentheses. 2. *** p<0.01, ** p<0.05, * p<0.1

The positive and statistically significant TIME coefficient suggests annually, the ratio of DI to GDP in food manufacturing increases by 0.09% ( Table 7). Thus, although the countries in the development groups have significantly different levels of DI, collectively, the DI is rising. This is in line with the notion that current years’ investments are explained by previous years’ investments ( Hall & Jorgensen, 1967). This contrasts with the negative coefficient for whole economies of developing countries ( Agosin & Machado, 2005) and developed countries ( Wang, 2010). Both the one- and two-year lags of growth of food manufacturing value-added coefficients suggest for unit increases GR_1 and GR_2, the DI ratio increases by at least 2%. This is expected as increased income from food manufacturing can be channelled into savings. This would then become investable funds for the sector. This result also confirms the theoretical position that the level of output is one of the drivers of the investment function ( Jorgenson, 1963). 

The dynamic effects of FDI on DI are presented in Table 8. The null hypothesis that the statistic of 0.0056 is different from zero could not be rejected. Thus, there is no short-run effect of FDI on DI in food manufacturing for developing countries. This result is supported by the evidence from the existing literature on the whole economies of individual economies and groups of economies ( Agosin & Machado, 2005; Ahmad *et al.,* 2018; Djokoto, 2013; Josue *et al.,* 2014; Oualy, 2019; Wang, 2010). The findings of Kim & Seo (2003) however, disagree with this result.

**Table 8.** Dynamic effects of FDI on DI for countries with different levels of development.

	Computed Statistic	Chi-square value	Effect
Developing economies			
Short run	0.0056	0.01	No effect
Long run	0.3711	9.16	Crowd-out
Economies in transition			
Short run	0.0466	2.28	No effect
Long run	0.6443	3.63	Crowd-out
Developed economies			
Short run	-0.0154	8.38	Crowd-out
Long run	0.6662	54.59	Crowd-out
Combined: Developing, transition and developed economies			
Short run	0.0676	1.08	No effect
Long run	1.7731	8.09	Crowd-in

In the case of the long run, the null hypothesis that the Wald statistic value of 0.3711 is indistinguishable from 1 was rejected at a chi-square value of 9.16. Since the statistic is less than 1, there is a crowd-out effect in the long run for developing country food manufacturing. For a one-dollar increase in FDI in food manufacturing, DI in the food manufacturing sector increases by 0.37 (less than one dollar). This is like the findings of Agosin & Machado (2005) for Latin America, Budang & Hakim (2020) for Asia, Morrissey & Udomkerdmongkol (2012) for developing countries, Mutenyo & Asmah (2010) for sub-Saharan Africa countries, and Oualy (2019) for Cote d’Ivoire and Babu (2021) for India. Josue *et al.* (2014) and Kim & Seo (2003) however, found a neutral effect whilst Wang (2010) and Farla *et al.* (2016) reported a crowd-in effect.

For economies in transition, the results are akin to that of the developing countries; no effect in the short run and crowd-out effect in the long run. The exception is that the statistics are larger than those of the developing countries. This suggests the extent of crowding out is less severe for transition economies than for developing economies. In the case of the long run, one-dollar increase in FDI will lead to 0.64 dollars (less than one dollar) increase in DI in the food manufacturing sector. Whilst the short-run result agrees with Jude (2019) and Kejžar (2016), it departs from that of Mileva (2008), who found a crowd-in effect for transition economies. However, in the long run, Mileva (2008) found a neutral effect for transition economies. 

Turning to developed countries, there is crowd-out for both short-run and long-run effects. For the latter effect, one dollar increases in FDI into food manufacturing results in a decrease of 0.015 dollars in DI. Crowd-out estimates with negative values are more detrimental than those with positive values. In the case of the former, there is an actual decrease in DI whilst for the latter, the magnitude is an increase *albeit* less than one. The short-run crowd-out found for food manufacturing in developed countries agrees with that found by Wang (2010) for the whole economy of developed countries. Whilst the short-run result departs from those of the developing and transition economies, that for the long run is similar. Further, the coefficient for the long run is slightly higher than that of economies in transition. This result is like that of Mišun & Tomšík (2002) for Poland but departs from the Czechia and Hungary results from Mišun & Tomšík (2002); Pilbeam & Oboleviciute (2012) for EU-12 who reported crowd-in effects and the neutral effect for developed economies by Wang (2010). 

Some reasons can be adduced for the long-run crowd-out effects. In the case of developed economies, over the study period, they have witnessed significant mergers and acquisitions (M&A). This can be situated within development and FDI theories ( Dunning, 1977; Dunning, 1988; Dunning, 2001; Intriligator, 2004; Stiglitz, 2004a; Stiglitz, 2004b; Tanzi, 2004). Transfer of assets from domestic firms to MNEs means loss of the record of DI in food manufacturing particularly in cases where proceeds from acquisitions are not channelled into new food manufacturing facilities. Thus, M&As by foreign firms in host countries could also lead to crowd-out effects although not so much the case for greenfield investments ( Ashraf & Herzer, 2014; Punthakey, 2020; UNCTAD, 1998). Secondly, MNEs are known to also set up sister companies within the host country or import inputs from affiliate and non-affiliated companies abroad ( Gallova, 2011). This deprives host country firms’ customs of the MNEs in the host country.

For developing economies in transition, the above and other reasons account for the long-run effects. First, developing and transition countries do engage in food manufacturing using domestic resources and investments. Attracting foreign investment presupposes local investments are inadequate based on the existing policy environment and conditions. Therefore, there will be a need for more attractive conditions to woo foreign investment. As a result, foreign investors tend to enjoy some benefits which may not be available to local investors.

Second, is the mode of entry; mergers and acquisitions. Through M&As, the original business ceases to exist. In cases where the previous shareholders do not re-invest in food manufacturing, this could result in a crowd-out of domestic investment. Third, foreign firms could import inputs from affiliate and non-affiliated companies abroad ( Gallova, 2011). Such actions reduce market opportunities for host country firms. Not only do these firms fail to increase output and thereby re-invest, but also lose the market. This could result in complete shutdowns. 

Fourth, is the macroeconomic dimension. Domestic currency can depreciate partly due to periodic profit transfers by MNEs and the opening of the financial markets. Local firms that do not export would have difficulty remaining in business as re-investments will become more expensive ( Desai *et al.,* 2004). The fifth is the weak investment regulation environment in some countries ( Ufimtseva, 2020). Whilst these could lead to delays in translating FDI on the balance of payments into investments, the weak investment regulation environment could have other effects. Failure to ensure MNEs comply with domestic investment guidelines would lead to delay in realising the effects of FDI in the host economy; job creation that would lead to increases in income that would transmit to savings and consequently investments. MNEs could flout requirements for joint ownership arrangements. 

Finally, lower managerial acumen, consequent lower performance of local firms than foreign firms, and failure of domestic firms to update technology could make it difficult for host country firms to become competitive hence, could be crowded out ( Djokoto, 2013; UNCTAD, 2015). This reflects the competition effect ( Markusen & Venables, 1999). 

Combining all three development groups, the short-run effect coincides with those of developing and transition economies; no significant effect. The effects for the latter two certainly influenced the short-run effect for all three economy groups more than that of the former, developed economies. Statistical and economic and administrative reasons can be adduced to explain the overall short-run results. The standard errors of the long-run statistics are high relative to the coefficients for the short run, in some cases, covering the coefficients almost two times. This accounted for the insignificant effect of FDI on DI in the short run for developing economies in transition. From the economics and administrative perspective, resource acquisition in the host country could take some time to materialise just like regulatory and administrative processes. In the same vein, the setting up of processing facilities could last more than a year. Therefore, multinational enterprises may fail to transform all FDI into investment in the sense of the national account ( Agosin & Machado, 2005). Thus, the economic effects of FDI on DI have a transmission trajectory that can only be transcended over lapsed time. In developing and transition economies where there could be significant regulatory and administrative inefficiencies, these time lags can be pronounced. In light of these, statistically significant short-run effects of FDI on DI are unlikely. 

In the long run, one dollar increase in FDI into food manufacturing leads to 1.7731 dollars (more than one dollar) increase in DI. It is the crowd-in effect for the food manufacturing sector jointly. The long-run result agrees with the findings of Jude (2019) and Kejžar (2016) for transition economies, Pilbeam & Oboleviciute (2012) for EU12 and Wang (2010) for least developed countries. Although the long-run crowd-in effect is desirable, the result departs from the long-run crowd-out effect for each of the development groups. The departure, from the development groups, is rather interesting yet plausible. As the computation of the long-run effect is not an arithmetic average for which there should be a less than 1.00 value, but a statistical summation of the interaction of the coefficients with the standard errors (the Wald statistic), the more than 1.0 value is an acceptable outcome. Six out of the nine coefficients of FDI were positive. However, a closer examination revealed that the sizes of the positive coefficients were far larger than that of the negative coefficients. The DI coefficients were similar, two coefficients had negative signs but the other four had positive signs. Together, the size of the positive coefficients exceeds that of the negative coefficients. The effects of the DIs result in a small increase in the sum of the DI. Adding these to the coefficients of FDIs in Equation 10, produced a value greater than 1. The departure from the economic grouping results also confirms the approach of the paper to segregate the analysis into levels of development. Indeed, the total sample results would have masked the group results that would have led to a one-size-fits-all recommendation. Thus, different policy propositions will be required for the various development groups.

The overall crowd-in effect can be explained in the literature. Foreign direct investment is known to promote knowledge, technology transfer, exports expansion, product and process innovation ( Djulius, 2017; Jawaid *et al.,* 2016; Jin *et al.,* 2019; Mohanty & Sethi, 2019; Schneider & Wacker, 2020; Viglioni & Calegario, 2020). Whilst employees could share knowledge on the job, labour mobility within the food manufacturing sector (from MNEs to domestic firms), would also lead to knowledge diffusion. Technology transfer through technology transfer agreements and licensing ( Hymer, 1976) creates an opportunity to induce investment in the domestic economy. Undoubtedly, the entry of MNEs creates some level of competition. Competition in output markets and observation and imitation of FDI in food manufacturing would lead to expansion of existing businesses, and the establishment of new business ventures ( Abebe *et al.,* 2019). Competition could also compel local firms to develop niche markets for their products. The utilisation of products from MNEs could also be sources of new businesses locally ( Alfaro, 2015; Santacreu-Vasut & Teshima, 2016). Indeed, linkage effects and the spillover effect of FDI are plausible ( Barro & Sala-i-Martin, 2004).

## Conclusion

This paper fills a void in the literature by investigating the effect of FDI on DI in the food manufacturing sector for developing, economies in transition and developed countries. Using an unbalanced panel data of 47 countries from 1993 to 2016, estimated by the system GMM, and computation of the Wald statistics from key terms including those with interactions, some findings were made. 

Developed economies experienced a crowd-out effect of FDI on DI in the short run, whilst the others experienced no significant effect. In the case of the long run, whilst food manufacturing sectors of all countries at the three levels of development separately exhibit crowd-out effects, the less than one increase in DI is smaller from the direction of developed-transition-developing countries. Level of development thus moderates the crowd-out effect in the food manufacturing sector. The effect in the long run for all development groups together is a crowd-in.

Developing economies and those in transition need to improve regulatorily and administrate efficiency. Specific attention to improving the ease of doing business and reducing time to register new businesses would be helpful. Automation of processes and the use of computer web applications for business transactions would be useful. These would minimise human interface and reduce delays as well as reducing opportunities for rent-seeking.

Review of investment policies and strengthening of the enforcement regimen would ensure improved compliance with the investment regulations, technology transfer, payment of appropriate taxes among others. Investment policies should prioritise partnerships (linkages) and mergers over complete acquisitions. Encouraging MNEs to engage in export through the Free Zones concept would promote exports and increase foreign exchange. This would ease the pressure on the local currency generally and especially during periods of profit repatriation. 

For further research, the cost of capital can be considered in the theoretical model as capital markets improve across the globe, leading to the effectiveness of the role of interest rates in determining domestic investment and the availability of reliable data.

## Data availability

### Underlying data

Data have been obtained from the Food and Agriculture Organization of the United Nations: http://www.fao.org/faostat/en/#data

Figshare: Foreign Direct Investment crowding out Domestic Investment in Food Manufacturing, https://doi.org/10.6084/m9.figshare.13591235.v2 ( Djokoto, 2021).

Data are available under the terms of the Creative Commons Zero "No rights reserved" data waiver (CC0 1.0 Public domain dedication).

## Notes

^1^ The estimates include tobacco.

^2^ Agosin & Machado (2005) proposed ${\hat{\beta }}_{LT}=\frac{\sum_{j=3}^{5}{\hat{\beta }}_{j}}{1-\sum_{j=1}^{2}{\hat{\beta }}_{j}}=1.$ Multiplying both sides of the equation by $1-\sum_{j=1}^{2}{\hat{\beta }}_{j}$ and adding $\sum_{j=1}^{2}{\hat{\beta }}_{j}$ to both sides yields Equation 7, Equation 8, Equation 9 and Equation 10.
