# Supplementary material for: Level of development, foreign direct investment and domestic investment in food manufacturing
Source: F1000Res. 2022 Jul 1;10:72. Originally published 2021 Feb 4. [Version 3] doi: 10.12688/f1000research.28681.3 (PMC10039321; doi:10.12688/f1000research.28681.3)
Supplement: Peer review history

# Peer review: Reviewer response for version 3

**DOI:** 10.5256/f1000research.135534.r162939

**Published:** 2023-03-24

**Authors:** Novica Supic

**Affiliations:** 1 Faculty of Economics, University of Novi Sad, Novi Sad, Serbia

In general, the paper is well-written; however, there are some concerns that need to be addressed before accepting it for indexing. The presentation of relevant theories is overly extensive, as numerous theories are presented with minimal explanation of the mechanisms of FDI influence, which is the main focus of the work. As such, it is necessary to provide a more detailed presentation of the theories referenced by the author in the section where the obtained results are discussed.

 Additionally, it is important to explain why variables with a time lag of t-2 are used as explanatory variables in the regression, alongside the time lag of t-1. What is the criterion for choosing a lag of t-2 instead of, for instance, a lag of t-3? The paper lacks unit root tests in panel models and tests for cross-section dependence.

 Furthermore, the recommendations for public policymakers are overly general and do not clearly stem from the results of the econometric analysis or the theories presented.

Is the work clearly and accurately presented and does it cite the current literature?

Yes

If applicable, is the statistical analysis and its interpretation appropriate?

Yes

Are all the source data underlying the results available to ensure full reproducibility?

Yes

Is the study design appropriate and is the work technically sound?

Yes

Are the conclusions drawn adequately supported by the results?

Yes

Are sufficient details of methods and analysis provided to allow replication by others?

Partly

Reviewer Expertise:

Income inequality, Poverty, Economic policy, FDI

I confirm that I have read this submission and believe that I have an appropriate level of expertise to confirm that it is of an acceptable scientific standard, however I have significant reservations, as outlined above.

# Peer review: Reviewer response for version 3

**DOI:** 10.5256/f1000research.135534.r142975

**Published:** 2022-07-04

**Authors:** Jean Michel Roy Oualy

**Affiliations:** 1 Università degli Studi di Macerata, Macerata, Italy

I confirm my ‘Approved’ status.

 Congratulations to the authors.

Is the work clearly and accurately presented and does it cite the current literature?

Yes

If applicable, is the statistical analysis and its interpretation appropriate?

Partly

Are all the source data underlying the results available to ensure full reproducibility?

Yes

Is the study design appropriate and is the work technically sound?

Yes

Are the conclusions drawn adequately supported by the results?

Yes

Are sufficient details of methods and analysis provided to allow replication by others?

Yes

Reviewer Expertise:

NA

I confirm that I have read this submission and believe that I have an appropriate level of expertise to confirm that it is of an acceptable scientific standard.

# Peer review: Reviewer response for version 2

**DOI:** 10.5256/f1000research.55226.r85181

**Published:** 2022-06-24

**Authors:** Festus Victor Bekun

**Affiliations:** 1 Faculty of Economics Administrative and Social Sciences, Istanbul Gelisim University, Istanbul, Turkey

The topic is interesting, and the author has done a great job in realizing the subject. However, there are few areas on the paper that is still lagging and should be addressed properly.

 Abstract

1. The authors should motivate the choice of variables with theory and empirical backing on the subject
2. Keywords should be revised to match key element of title
3. Rewrite the title to be more catchy

Introduction

 The objective of the paper presented needs more clarifications to suit reader to understand the main idea of the paper especially for the study case is needed

 Literature review

 The literature is well written. However, there is need for more recent studies ranging from 2018-2022 to motivate the study properly. The entire study is too scanty and the related literature is not exhausted

 Revisiting the Nexus between FDI, financial development and economic growth: Empirical evidence from Nigeria. *Journal of Public Affairs*, e2561 ^1^.

 The effects of air transportation, energy, ICT and FDI on economic growth in the industry 4.0 era: Evidence from the United States. *Technological Forecasting and Social Change*, *160*, 120297 ^2^.

 Trade openness, FDI, and income inequality: Evidence from sub‐Saharan Africa. *African Development Review*, *33*(1), 193-203 ^3^.

 Mitigating emissions in India: accounting for the role of real income, renewable energy consumption and investment in energy. 12(1), pp. 188-192 ^4^.

 Methodology

 This section is generally well motivated, Kindly take note of the following minor additions:

1. More benefit of the various techniques utilized should be stated
2. Check for cross-sectional dependency and add correlation text and VIF
3. The authors should avoid much mathematical expressions or take some to appendix and make the study reader friendly for other practitioners other than academic without compromise for study intent and quality.

Discussion

1. The discussion is well written, but the authors should link their findings to the previous studies in the literature.
2. There is need for professional proofreading or consult English native support

Conclusion

1. The sub-title should be conclusion and policy recommendation not only conclusion
2. The policy which is the engine of the study is weak and small. I therefore encourage the authors to elaborate more on the policy recommendations to policy makers for the investigated bloc

The authors should add limitations of the study and future recommendation.

Is the work clearly and accurately presented and does it cite the current literature?

Partly

If applicable, is the statistical analysis and its interpretation appropriate?

Partly

Are all the source data underlying the results available to ensure full reproducibility?

Partly

Is the study design appropriate and is the work technically sound?

Partly

Are the conclusions drawn adequately supported by the results?

Partly

Are sufficient details of methods and analysis provided to allow replication by others?

Partly

Reviewer Expertise:

NA

I confirm that I have read this submission and believe that I have an appropriate level of expertise to confirm that it is of an acceptable scientific standard, however I have significant reservations, as outlined above.

## References

[1] : Revisiting the Nexus betweenFDI , financial development and economic growth: Empirical evidence from Nigeria. *Journal of Public Affairs* .2020; 10.1002/pa.2561 10.1002/pa.2561

[2] : The effects of air transportation, energy, ICT and FDI on economic growth in the industry 4.0 era: Evidence from the United States. *Technol Forecast Soc Change* .2020;160: 10.1016/j.techfore.2020.120297 120297 10.1016/j.techfore.2020.120297 32934425PMC7484704

[3] : Trade openness, FDI, and income inequality: Evidence from sub‐Saharan Africa. *African Development Review* .2021;33(1) : 10.1111/1467-8268.12511 193-203 10.1111/1467-8268.12511

[4] : Mitigating Emissions in India: Accounting for the Role of Real Income, Renewable Energy Consumption and Investment in Energy. *International Journal of Energy Economics and Policy* .2022;12(1) : 10.32479/ijeep.12652 188-192 10.32479/ijeep.12652

# Response

**Published:** 2022-06-24

**Authors:** Justice Djokoto

**Affiliations:** Central University, Ghana

Abstract

**Comment**

- The authors should motivate the choice of variables with theory and empirical backing on the subject

**Response**

 These have already been accomplished for the whole model in the methods section.

**Comment**

- Keywords should be revised to match key element of title

**Response**

 Since keywords are used in search engines together with the titles, using words and phrase same as those in the title is not advisable. Rather, the keywords should reflect other words that convey the content to the study that have not been used in the title.

**Comment **

- Rewrite the title to be more catchy

**Response**

 The paper has been published in Feb 2021 and has already been cited 7 times in google scholar. Re-writing the title at this stage be unhelpful. Moreover, the construction of the title does reflect the content of the work.

**Comment**

 Introduction

 The objective of the paper presented needs more clarifications to suit the reader to understand the main idea of the paper especially for the study case is needed

**Response**

 The objective is simple: “This paper fills these gaps by investigating the effect of FDI on DI in the food manufacturing sector for developing, economies in transition and developed countries.” This can be found in the third paragraph of the ‘Introduction’.

**Comment**

 Literature review

 The literature is well written. However, there is a need for more recent studies ranging from 2018-2022 to motivate the study properly. The entire study is too scanty and the related literature is not exhausted.

**Response**

 The manuscript was prepared in the year 2020 and was submitted on the 17 ^th^ of December 2020. This contained many references dating year-2020. ****Version 1 was published on 4 ^th^ February 2021. For the empirical literature, the search terms “domestic investment” AND “foreign direct investment’ were used. We applied this search term with the 2021 date. This brought only 1 result:

 “Babu, A. (2021). Foreign Direct Investment--Domestic Investment Nexus: Evidence from India. *Contemporary Economics*, *15*(3), 267-276.” This is used to update the paper.

 Interestingly, none of the suggested literature popped up:

1. Olorogun L, Salami M, Bekun F: Revisiting the Nexus between FDI, financial development and economic growth: Empirical evidence from Nigeria. *Journal of Public Affairs*. 2020. 
  2. Adedoyin FF, Bekun FV, Driha OM, Balsalobre-Lorente D: The effects of air transportation, energy, ICT and FDI on economic growth in the industry 4.0 era: Evidence from the United States. *Technol Forecast Soc Change*. 2020; 160: 120297
  3. Xu C, Han M, Dossou T, Bekun F: Trade openness, FDI, and income inequality: Evidence from sub‐Saharan Africa. *African Development Review*. 2021; 33 (1): 193-203 
  4. Bekun F: Mitigating Emissions in India: Accounting for the Role of Real Income, Renewable Energy Consumption and Investment in Energy. *International Journal of Energy Economics and Policy*. 2022; 12 (1): 188-192 

A critical examination of the suggested literature showed that 1 – 3 contained ‘foreign direct investment’. But this was not related to ‘domestic investment’ in line with our search terms. Regarding paper 4, both the title and the keywords did not contain either ‘foreign direct investment’ or ‘domestic investment’. Further, there was no indication of ‘food manufacturing’. Consequently, the whole text was searched and ‘foreign direct investment’ or ‘domestic investment’ were not found. Hence, the suggested literature is not relevant to the context of the study’s literature.

**Comment**

 Methodology

 This section is generally well-motivated, Kindly take note of the following minor additions:

- More benefits of the various techniques utilized should be stated

**Response**

 These have been added.

**Comment**

- Check for cross-sectional dependency and add correlation text and VIF

**Response**

 Correlations of key variables are already in Table 4.

**Comment**

- The authors should avoid many mathematical expressions or take some to the appendix and make the study reader-friendly for other practitioners other than academics without compromising for study intent and quality.

**Response**

 The equation 1 - 10 are basic to the study. Equation 2 is the estimated model whilst equations 3-10 show how the effects are obtained. I have elected to keep them in their place.

**Comment**

 Discussion

- The discussion is well written, but the authors should link their findings to the previous studies in the literature.

**Response**

 The findings are linked to previous research. The previous research is in coloured text in the discussion section.

**Comment**

- There is a need for professional proofreading or consulting English native support

**Response**

 This has been done.

**Comment**

 Conclusion

- The sub-title should be conclusion and policy recommendation not only conclusion

**Response**

 The section contains the policy recommendations. The use of the subtitle ‘Conclusion’ is in line with the author guidelines of F1000research.

**Comment**

- The policy which is the engine of the study is weak and small. I, therefore, encourage the authors to elaborate more on the policy recommendations to policymakers for the investigated bloc

**Response**

 Two paragraphs have been devoted to the policy recommendations.

# Peer review: Reviewer response for version 1

**DOI:** 10.5256/f1000research.31726.r78909

**Published:** 2021-02-26

**Authors:** Jean Michel Roy Oualy

**Affiliations:** 1 Università degli Studi di Macerata, Macerata, Italy

- Which econometric model and economic theory are used?
- What are the different econometrics and statistical tests used to write the equation (1)?
- Explain why you take a linear model with lagged variables and with all countries together.
- Which econometric model and economic theory is used to write the equations (2), (3), (4), (5), (6), (7), (8), (9), and (10)? I suggest you add external instruments.
- I suggest you add external instruments.

Is the work clearly and accurately presented and does it cite the current literature?

Yes

If applicable, is the statistical analysis and its interpretation appropriate?

Partly

Are all the source data underlying the results available to ensure full reproducibility?

Yes

Is the study design appropriate and is the work technically sound?

Yes

Are the conclusions drawn adequately supported by the results?

Yes

Are sufficient details of methods and analysis provided to allow replication by others?

Yes

Reviewer Expertise:

NA

I confirm that I have read this submission and believe that I have an appropriate level of expertise to confirm that it is of an acceptable scientific standard.

# Response

**Published:** 2021-02-27

**Authors:** Justice Djokoto

**Affiliations:** Central University, Ghana

**AUTHOR RESPONSE TO REVIEWER COMMENTS**

*Comment*

 Which econometric model and economic theory are used?

*Response*

*The econometric model is the system GMM. The Agosin and Machado (2005) model is based in the neoclassical investment theory.*

*Comment*

 What are the different econometrics and statistical tests used to write the equation (1)?

*Response*

*Equation 1 was adapted from Agosin and Machado (2005) that theoretically derived the model which has been widely used. The estimation of equation 1 has been subjected to econometric and statistical tests outlined in the section ‘Estimation procedure’. The pieces of evidence of the tests are shown in Table 3 – 7.*

*Comment * 

 Explain why you take a linear model with lagged variables and with all countries together.

*Response*

1. *Agosin and Machado (2005) who derived the model used linear estimations. *
2. *Other authors did the same. *
3. *The lag variables and the possibility of endogeneity of the explanatory variables were recognised by estimating the system GMM. *
4. *The countries were segregated into development groups, developing, developed, transition. * 

*Comment*

 Which econometric model and economic theory is used to write the equations (2), (3), (4), (5), (6), (7), (8), (9), and (10)? I suggest you add external instruments.

*Response*

*Regarding equation 1, the econometric model is the system GMM. The Agosin and Machado (2005) model is based on the neoclassical investment theory. The nature of the data, comprising developing, developed and the transition was accounted for in equation 2. Equation 3 to 6 presents the Wald computation of the short-run effects of FDI on DI for the respective development groups as well as the combined set of country groups. Equation 7 to 10 presents the Wald computation of the long-run effects of FDI on DI for the respective development groups as well as the combined set of country groups.*

*During the estimation, the appropriate instrument was selected. This was not the same for all the estimated models reported hence the decision to exclude it in the model specification.*

*Comment* 

 I suggest you add external instruments.

*Response*

*The instruments which were various levels of lags differed for each of the model estimations reported. Hence the decision to exclude it in the model specification as the model specification is general.*
